# Benzylglucosinolate Derived Isothiocyanate from *Tropaeolum majus* Reduces Gluconeogenic Gene and Protein Expression in Human Cells

**DOI:** 10.1371/journal.pone.0162397

**Published:** 2016-09-13

**Authors:** Valentina Guzmán-Pérez, Christiane Bumke-Vogt, Monika Schreiner, Inga Mewis, Andrea Borchert, Andreas F. H. Pfeiffer

**Affiliations:** 1 Department of Clinical Nutrition, German Institute of Human Nutrition, Potsdam-Rehbrücke, Nuthetal, Germany; 2 Department of Nutrition and Biochemistry, Sciences Faculty—Pontificia Universidad Javeriana, Bogotá D.C, Colombia; 3 Department of Endocrinology, Diabetes and Nutrition, Charité- Universitätsmedizin Berlin, Berlin, Germany; 4 Department of Plant Quality, Leibniz-Institute of Vegetable and Ornamental Crops Großbeeren/Erfurt e.V, Erfurt, Germany; National Institutes of Health, UNITED STATES

## Abstract

Nasturtium (*Tropaeolum majus* L.) contains high concentrations of benzylglcosinolate. We found that a hydrolysis product of benzyl glucosinolate—the benzyl isothiocyanate (BITC)—modulates the intracellular localization of the transcription factor Forkhead box O 1 (FOXO1). FoxO transcription factors can antagonize insulin effects and trigger a variety of cellular processes involved in tumor suppression, longevity, development and metabolism. The current study evaluated the ability of BITC—extracted as intact glucosinolate from nasturtium and hydrolyzed with myrosinase—to modulate i) the insulin-signaling pathway, ii) the intracellular localization of FOXO1 and, iii) the expression of proteins involved in gluconeogenesis, antioxidant response and detoxification. Stably transfected human osteosarcoma cells (U-2 OS) with constitutive expression of FOXO1 protein labeled with GFP (green fluorescent protein) were used to evaluate the effect of BITC on FOXO1. Human hepatoma HepG2 cell cultures were selected to evaluate the effect on gluconeogenic, antioxidant and detoxification genes and protein expression. BITC reduced the phosphorylation of protein kinase B (AKT/PKB) and FOXO1; promoted FOXO1 translocation from cytoplasm into the nucleus antagonizing the insulin effect; was able to down-regulate the gene and protein expression of gluconeogenic enzymes; and induced the gene expression of antioxidant and detoxification enzymes. Knockdown analyses with specific siRNAs showed that the expression of gluconeogenic genes was dependent on nuclear factor (erythroid derived)-like2 (NRF2) and independent of FOXO1, AKT and NAD-dependent deacetylase sirtuin-1 (SIRT1). The current study provides evidence that BITC might have a role in type 2 diabetes T2D by reducing hepatic glucose production and increasing antioxidant resistance.

## Introduction

Type 2 diabetes (T2D) is a health problem throughout the world [1]. T2D is characterized by insulin resistance, which leads to hyperglycemia, owing at least in part to the impaired ability of insulin to suppress expression or activity of gluconeogenic enzymes [2]. In T2D an increase in the production of free radicals with a subsequent induction of oxidative stress is also present [3]. Under oxidative stress conditions the insulin signaling is reduced, which may contribute to insulin resistance, and to the progression of diabetes and related complications [4–6]. The presence of reactive oxygen species (ROS) activate the forkhead box O (FOXO) transcription factors. They mediate the effects of ROS through the modulation of gene transcription factors involved in several cellular processes including glucose metabolism, cell cycle arrest, antioxidant response and apoptosis [7], alterations in FOXO function could contribute to metabolic disorders in diabetes [8]. In humans, FOXO subgroup contains four members: FOXO1, FOXO3a, FOXO4 and FOXO6 [4]. Since FOXO1 has a variety of cellular functions, in some cases antagonistic, it is tightly regulated by external stimuli. Environmental signals, including insulin, growth factors, nutrients, cytokines and oxidative stress induce post-translational modifications, mainly phosphorylation, acetylation, mono- and poly-ubiquitination which regulate the levels, subcellular localization, and transcriptional activity of FOXO1 [9].

The translocation of FOXO1 from cytoplasm to the nucleus is mandatory for its transactivation, which modulates FOXO1 dependent transcription [9]. In the presence of insulin FOXO1 is negatively regulated by AKT/PKB induced-phosphorylation, which causes the sequestration of FOXO1 in the cytoplasm, thereby preventing FOXO1 from transactivating its target genes in the nucleus [10]. In the absence of insulin stimulation, during oxidative stress or in the fasting state, FOXO1 may induce oxidative stress resistance through the expression of the anti-oxidant enzymes manganese superoxide dismutase (MnSOD) and catalase (CAT) [11] and glucose production through the gene expression of phosphoenolpyruvate carboxykinase (PEPCK) and glucose-6-phosphatase (G6ase) [12]. This metabolic process can be regulated by mitogen-activated protein kinase phosphatase-3- (MKP-3) mediated dephosphorylation of FoxO1 at Ser256, which promotes its nuclear import and subsequent recruitment to the promoters of key genes [13]. Phytochemicals, such as the stilbene resveratrol and flavonoids like apigenin and luteolin have been shown to induce FOXO1 nuclear accumulation and activation as well, and to promote the gene expression of antioxidant enzymes [11, 14, 15].

Diet plays an important role in the prevention and management of T2D [16] and epidemiological and animal studies have shown that the consumption of some vegetables can delay or prevent the development of the disease [17]. The evidence for individual dietary components is scarce, but phytochemicals, a large group of secondary metabolites of plants used in nutrition, are thought to play a significant role in the health effects of plant-based diets, although the underlying mechanisms of these effects are still unclear [18].

Generally, brassicaceous plants such as *Brassica* vegetables (e.g. broccoli, cabbage, brussels sprouts, cauliflower) are marked by a specific group of phytochemicals–the glucosinolates [19]. More than 150 different naturally occurring glucosinolates have been recognized so far, although their content varies among different vegetables [20]. The Brassicales species nasturtium (*Tropaeolum majus*) is characterized especially by high concentrations of the predominant aromatic benzyl glucosinolate (1000 mg/100 g fresh matter) [20]. Glucosinolates itself are not the actual bioactive compounds, but the evidence shows that their corresponding hydrolyses products, especially isothiocyanates (ITCs), are potent inducers of phase II detoxifying enzymes and subsequently confer protection against oxidative stress and chronic inflammation [21].

Despite of the modulatory effect of isothiocyanates on cytoprotective enzymes and inflammatory processes, only few studies have linked them with T2D prevention [22]. Recently, sulforaphane (4-methylsulfinylbutyl ITC) supplementation extracted from broccoli sprouts was shown to reduce insulin, inflammatory markers and LDL levels in T2D patients [22–24], although the mechanisms underlying these effects are not clear. Also *Moringa* extracts supplementation rich in 4(α-l-rhamnosyloxy)-benzyl isothiocyanate was shown to display anti-obesity and anti-diabetic properties increasing insulin signaling and sensitivity [25, 26]. According to the European Prospective Investigation into Cancer and Nutrition (EPIC) the average consumption of aromatic glucosinolates as benzyl glucosinolate varies between the European countries: 1.4 mg/d (men) to 0.74 mg/d (women) in German population [27], and 4.46 mg/d (men) to 4.06 mg/d (women) in Spanish population [28], suggesting differently high, but regular intake of Brassicales species.

Since the PI3/AKT/FOXO1 pathway has a critical role in the metabolic action of insulin, and FOXO1 is the mediator for gene expression alteration by insulin [29], we developed a fluorescence microscopic FOXO1- green fluorescent protein (FOXO1-GFP) translocation assay to clarify the anti-diabetic properties of ITCs. Therefore, this study evaluated the ability of BITC, extracted from the edible plant *Tropaeolum majus*, to modulate the insulin-signaling pathway and their effects on i) the intracellular localization of FOXO1 and ii) the expression of proteins involved in glucose metabolism and antioxidant response. The present study provides detailed insights into the effect of BITC on the PI3K/AKT/FOXO1 pathway, as BITC is responsible for FOXO1 nuclear import and accumulation. BITC effect contributes to the antioxidant enzymes gene expression modulation and gluconeogenesis inhibition, possibly through the interaction with different nuclear transcription factors from the evaluated in the present study.

## Materials and Methods

### Cell lines and culture

Cell culturing of human osteosarcoma cell line (U-2 OS) and human hepatocellular carcinoma cell line (HepG2) was carried out under humid atmosphere, at 37°C with a 5% CO_2_ content. The cells were purchased from ECACC (European Animal and Cell Collection). Due to their more extended morphology U-2 OS cells were used for fluorescence-microscopic analyses to evaluate the effect of BITC on FOXO1 translocation. HepG2 cells were selected to evaluate the effect of BITC on gene and protein expression including liver specific gluconeogenic gene expression. HepG2 were cultivated in EMEM with 1g glucose/L, low level like under conditions of starvation before a meal in humans; stable glutamine; 2.2 g NaHCO3, supplemented with 1% NEAA (Non-Essential Amino acids) and 10% Fetal calf serum (FCS). U-2 OS were grown in DMEM with 4.5 g glucose/L like after a meal (for better growth); 3.7 g/L NaHCO3; stable glutamine and Na-pyruvate supplemented with 10% FCS. Media were proposed by ECACC (European Animal and Cell Collection) for cell cultivation.

U-2 OS were transfected with DNA plasmids using Lipofectamine 2000 in Opti-MEM® I Reduced-Serum Medium (Invitrogen) according to the manufacturer´s description. A stable transfection of U-2 OS for constitutive expression of green fluorescent protein (GFP) labelled FOXO1 was obtained using pEGFP-N1 as vector, with an insertion of FOXO1 cDNA (complete FOXO1 coding sequence (CDS) in front of the CDS of EGFP. The vector used for the transfection was a gift as courtesy from Terry Unterman (University of Illinois at Chicago, USA) [30].

Following transfection, cells were grown in DMEM plus 10% FCS. Geneticin G418 sulfate (Calbiochem) at 0.4 mg/ml was added after 48h, for selecting those genetically engineered cells carrying the plasmid with encoded resistance. Separating cells over 3 passages resulted in homogeneous cell colonies expressing FOXO1-GFP, monitored by fluorescence microscopic live cell imaging [15]. Prior cell incubation with test compounds, growth of sub-confluent U-2 OS cells was controlled for 16 h by an initial reduction from 10% FCS to 2% FCS, and further to 0% FCS in DMEM for 1h starvation.

For transfection of silencing RNA (siRNA) into HepG2 cells, combined DharmaFECT4 (D4) transfection reagent and siRNAs ON-TARGETplus SMARTpool siRNA (Dharmacon) were applied. SMARTpool siRNA contained four SMARTselection-designed siRNAs, pooled for efficient knock-down of each analyzed target: FOXO1, AKT, SIRT1, NRF2, and NON-Target siRNAs NT were used for control transfections according to the description of the manufacturer. Prior to cell incubation with test compounds HepG2 cells were starved for 16 h in EMEM with 1 g/L glucose without FCS. Stimulation of cells was performed with benzyl glucosinolate dissolved in FCS-free medium and treated with 100 μg myrosinase at 37°C during 15 min enabling the formation of BITC. Insulin stimulations were performed with 100 nM human insulin in FCS-free medium.

### Quantification of gene expression by qRT-PCR

Total RNA was extracted upon 24 h incubation with BITC and insulin, using the Nucleospin RNA II total isolation kit (Machery-Nagel). RNA purity and quantity were assessed using the NanoDrop 2000 UV/VIS spectrophotometer (Thermo Scientific). High-capacity cDNA RT-PCR Kit (Applied Biosystems) was used to reverse transcribe 1 μg of RNA. For quantitative real-time PCR (qRT), samples were diluted in DNase/RNase free water. A final volume of 5 μl (1 μl cDNA corresponding to 100 ng of total RNA), 2 μl SYBR® Green PCR Master Mix (Applied Biosystems), 300 nmol/L forward and reverse primer see Table 1. and 1.3 μl RNase/DNase free Water for each reaction was used. The measurement was performed with ViiA7 Real-Time PCR System (Life Technologies) with the following thermal cycler conditions: 10 min at 95°C followed by 40 alternated cycles of 15 sec at 95°C and 35 sec at 60°C. For melting curve analysis, the reactions were heated to 95°C for 15 sec, cooled down to 60°C for 15 sec and heated again to 95°C for 15 sec. Reactions were carried out in triplicates. The ribosomal protein L32 (RPL32) housekeeping gene was used for normalization as an endogenous control. The mean value was used for further calculations. Reported levels of mRNA are expressed as fold changes and compared to untreated control cells.

**Table 1:** Primer sequences for qRT-PCR.

Target	Oligo name	Oligo sequence (5' to 3') Fwd	Oligo sequence (5' to 3') Rev
60S ribosomal protein L32	hRLP-32	CAACGTCAAGGAGCTGGAAGT	TTGTGAGCGATCTCGGCAC
Forkhead box O1	hFOXO1	GGCTGGAAGAATTCAATTCGTC	ACCCTCTGGATTGAGCATCCAC
Glucose-6-phosphatase	hG6Pc	CCCCTGATAAAGCAGTTCCCT	ATACACCTGCTGTGCCCATG
nuclear factor (erythroid derived)-like2	hNRF2	AACTACTCCCAGGTTGCCCA	CAAGTGACTGAAACGTAGCCGA
Phosphoenolpyruvat-carboxykinase	hPEPCK	AAGTATGACAACTGCTGGTTGGC	ATAACCGTCTTGCTTTCGATCCT
Proteinkinase B	hAKT1	GCTTCTATGGCGCTGAGATTGT	TGATCTTAATGTGCCCGTCCTT
NAD-dependent deacetylase sirtuin-1 (Sirtuin1)	hSIRT1	ATGCTGGCCTAATAGAGTGGCA	CCTCAGCGCCATGGAAAAT
Catalase	hCAT	GCGGTCAAGAACTTCACTGAGG	GGTGTGAATCGCATTCTTAGGC
Glutathione peroxidase 2	hGPX-2	GTGCTGATTGAGAATGTGGC	AGGATGCTCGTTCTGCCCA
NAD(P)H dehydrogenase (quinone 1)	hNQO1	CATCACAGGTAAACTGAAGGACCC	CTCTGGAATATCACAAGGTCTGCG
Sulfiredoxin-1	hSRXN1	CTCAGTGCTCGTTACTTCATGGTC	GTTTGGCCCTTCCTCTTCCTCC
Superoxide dismutase	hSOD2	GCCTGCACTGAAGTTCAATGG	CGTTTGATGGCTTCCAGCA

### Protein extraction and Western blotting

Upon incubation with the compounds cells were lysed with lysis buffer (Cell Signaling Technology) supplemented with protease- and phosphatase-inhibitors (Roche). Each 20 μg of protein were denaturated in SDS-PAGE sample buffer at 95°C for 5 min and subsequently separated by sodium dodecyl sulfate (SDS)-polyacrylamide gel electrophoresis (PAGE). Separated proteins were transferred onto PVDF- nitrocellulose membranes (Immobilon-FL; Millipore) and analyzed for the presence of Phospho-FOXO1(Thr24)/FOXO3a (Thr32) and PEPCK (PCK2) with primary antibodies from Cell Signaling Technology and G6Pase-antibodies from Santa Cruz Biotechnology. For normalization alpha-Tubulin or Glyceraldehyde-3-phosphate dehydrogenase (GAPDH) antibodies from Cell Signaling Technology were used. Detection was performed with fluorescent secondary antibodies (LI-COR Biosciences for ODISEY) via the Odyssey Imaging System (Licor, Lincoln, NA).

### Path scan intracellular signaling array

A PathScan Signaling Array Kit (Cell Signaling Technology) with a fluorescent readout for the simultaneous detection of the phosphorylated signaling molecules protein kinase B: AKT (Thr308), AKT (Ser473), extracellular-signal-regulated kinase ERK1/2 (Thr202/Tyr204), and stress-activated kinases/c-Jun N-terminal kinase SAPK/JNK (Thr183/Tyr185) was used according to the manufacturer’s instructions. Cell lysates were obtained from HepG2 treated with BITC 30 μM, insulin 100 nM and insulin plus BITC for 30 min. Upon sonication and centrifugation, the clear supernatant was used for protein content determination. LI-COR Bioluminescence imager and ODYSSEY software were used for scans and analyses.

### Cell viability assay

The viability of HepG2 cells after 24 h incubation with selected BITC concentrations of 1 μM, 2 μM, 5 μM, 10 μM, 20 μM, 50 μM, and 100 μM was tested with the CellTiter96 Aqueous Cell Proliferation assay (Promega) according to the manufacturer’s instructions. The U-2 OS cells survival after BITC stimulation was observed during microscopic life cell imaging.

### FOXO-translocation assay

Stably transfected U-2 OS cells with pEGFP-FOXO1 expressing FOXO1 with C-terminal tagged GFP were used for FOXO1-GFP visualization by fluorescence microscopy with Zeiss “Axio Observer.Z1” inverted fluorescent microscope in black, clear bottom 96-well plates with poly-D-lysine coating (Becton, Dickinson and Company, USA). Life cell images were recorded every minute up to 1 h with a filter for GFP, for tracing intracellular translocation of FOXO1-GFP after cells treatment with: a) insulin 100 nM; b) BITC 30 to 100 μM; c) insulin + BITC; d) NAC (N-acetyl cysteine); e) BITC + NAC; f) insulin + NAC and g) BITC + insulin + NAC.

Staining with 4´,6-diamidino-2-paraphenylindole (DAPI) 200 nM (Invitrogen) was used for defining nuclear areas. Fluorescence microscopic UV-Exposition was performed in BD Pathway 435 Bioimager (Becton Dickinson and Company). Quantification of GFP signals in nuclear areas and cytoplasm was analyzed using BD AttoVision version 1.6/435. Ratios of GFP_nuclear intensity and GFP_cytoplasmic intensity as Ratio Nuc/Cyt were established. Ratios lower than 1 reflected a predominant cytoplasmic localization of FOXO1-GFP, while higher than 1 showed a nuclear accumulation of FOXO1. An induced translocation factor could be calculated after substance treatment by normalization versus untreated cells.

### Purification of glucosinolates for cell culture test systems and glucosinolate analysis

The benzyl glucosinolate was extracted from seeds of *Tropaeolum majus* (nasturtium) according to the method described in Lippmann *et al*. for aliphatic and aromatic glucosinolates [31]. Benzylglucosinolate was converted to benzylisothiocyanate after 15 minutes treatment with myrosinase (from Sinapis alba seed EC 3.2.1.147, purchased from Sigma-Aldrich Chemie GmbH). BITC recovery was more than 95% as deduced by Gas chromatography–mass spectrometry (GC-MS), while benzylglucosinolate could not be detected in High-performance liquid chromatography (HPLC) after 15 minutes incubation.

### Data handling and statistical analyses

One-way ANOVA with appropriate post hoc tests (Bonferroni and Dunnett’s Multiple Comparison post-hoc test) or 2-tailed unpaired *t* Test were used for statistical analysis using SPSS. Values of p ≤ 0.05 were assumed to be significant. All results are presented as means ± SEM. The half maximal effective concentrations EC50 and median inhibitory concentration IC50 values were calculated using GraphPad Prism 5.0 software (San Diego, CA, USA).

## Results

### BITC induces FOXO1 translocation in U-2 OS stably transfected with pEGFP-FOXO1

**Fig 1:**
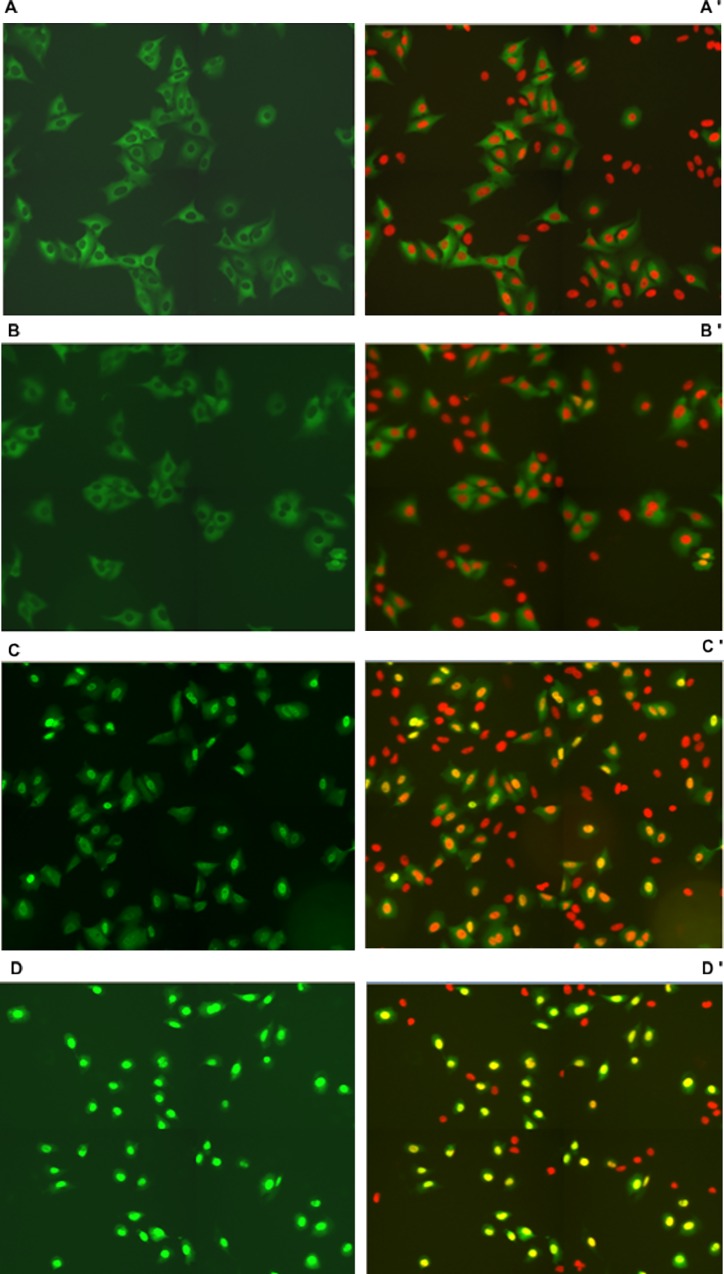
FOXO1 translocation in stably transfected U-2 OS (human osteosarcoma cells). Fig 1A–1D show FOXO1-GFP expressing cells and nuclear DAPI-staining (red) changing to orange-yellow in overlays with GFP after incubation with BITC at concentrations of 0 (1A-A’), 1 (1B-B’), 10 (1C-C’) and 100 μM (1D-D’), respectively. Following 1 h of starvation, stimulation of cells was performed with BITC for 1 h. Cells were fixed and stained with DAPI for identification of nuclear areas by fluorescence microscopic detection, for segmentation of cells and calculation of GFP-intensity ratios in nuclei and cytoplasm (Nuc/Cyt).

After treatment with BITC at concentrations of 1, 10 and 100 μM for 1 h translocation of FOXO1 was induced in stably transfected U-2 OS-FOXO1-GFP cell cultures in a dose dependent manner (Fig 1A–1D). Unstimulated cells showed FOXO1-GFP predominantly localized in the cytoplasm (Fig 1A).

**Fig 2:**
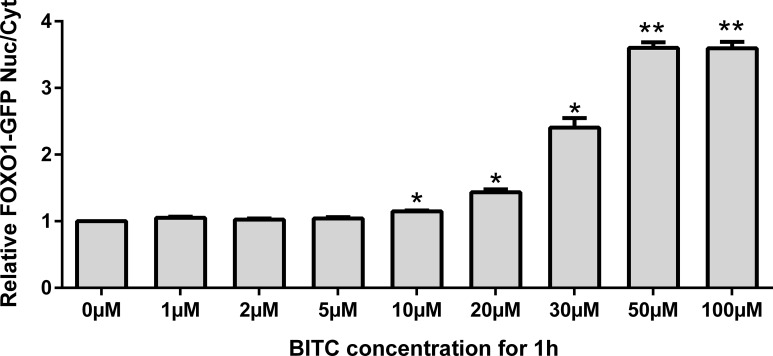
U-2 OS-FOXO1-GFP cells treated with BITC at selected concentrations. Nuclear accumulation was estimated by measuring the ratio of FOXO1 nuclear/FOXO1 cytosolic (FOXO1 Nuc/Cyt) after 1 h treatment normalized to untreated control cells. Ratios higher than 1 showed a nuclear accumulation of FOXO1. Results are presented as mean values + standard error (SEM) (n = 4). Significant differences vs control (0 μM) are labelled for *(p*<*0.05) and **(p*<*0.01) (Oneway ANOVA and posthoc Bonferroni multiple comparisons).

A significant FOXO1 nuclear accumulation with increasing BITC concentration was observed (Fig 2). A slight increase up to 1.14-fold was observed for BITC concentrations below 10 μM, becoming progressively more significant (up to 3.59-fold) for concentrations between 10 μM and 50 μM. Further increase in BITC concentration did not modify nuclear accumulation of FOXO1. Time-dependent FOXO1 translocation was also observed by life cell imaging of U-2 OS FOXO1-GFP in living cells during incubation for 1 h with BITC 50 μM. In control cells FOXO1 was located in cytoplasm but with increasing concentrations FOXO1 migrated to the nuclei (S1 Video).

**Fig 3:**
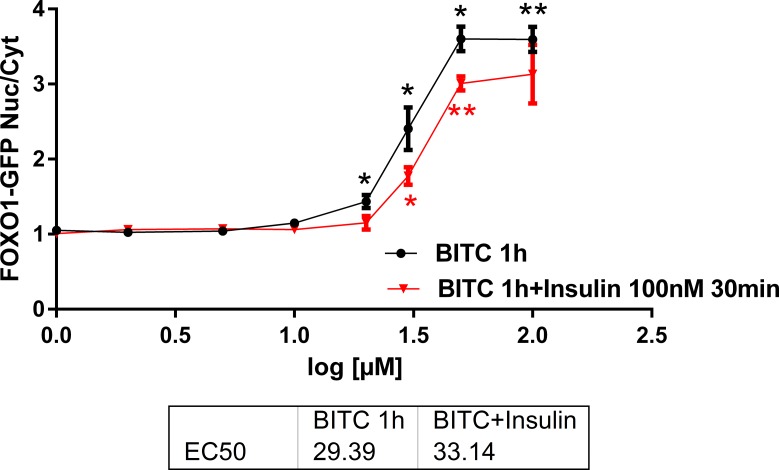
U-2 OS-FOXO1-GFP cells treated with BITC 1–100 μM for 1h and addition of 100 nM insulin after 30 min. The curves show the relative nuclei/cytoplasm intensity of FOXO1-GFP for BITC without (black curve) and with insulin addition (red curve). Results are presented as mean values + standard error (SEM) (n = 4). Significant differences vs control (0 μM) are labelled for *(p<0.05) and **(p<0.01) (Oneway ANOVA and posthoc Dunnett T3).

The BITC dose dependency was evaluated in the range 1 μM to 100 μM during 1h and the reversibility of FOXO1 translocation into nuclei was tested by adding 100 nM insulin for 30 min into parallel cell cultures (Fig 3). Insulin 100 nM applied 30 min after BITC partially reversed FOXO1 translocation induced by BITC 10 μM to 100 μM, which was significant for the doses 20 to 100 μM. The half maximal effective concentrations (EC50) values were 29.4 μM and 33 μM for BITC and BITC + insulin, respectively. A competition between nuclear import by BITC and export by insulin can be deduced from these data.

### FOXO1 translocation from cytoplasm into the nucleus is inhibited by the antioxidant N-acetyl-cystein (NAC)

**Fig 4:**
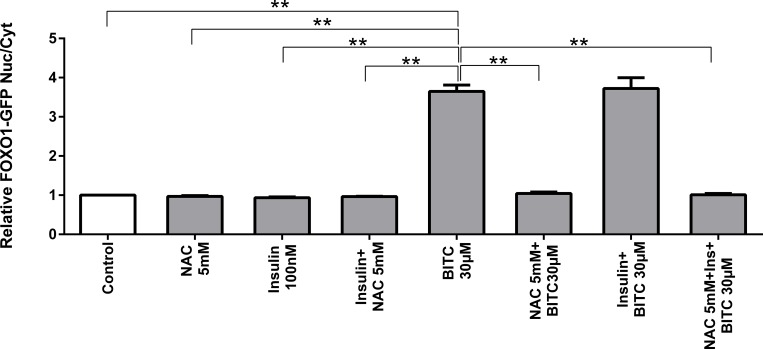
Inhibition of U 2-OS-FOXO1-GFP cells translocation upon antioxidant protection. Stimulation with insulin 100 nM, BITC 30 μM, insulin + BITC, NAC 5 mM (N acetyl cysteine), BITC + NAC, insulin + NAC and BITC + insulin + NAC for 2 h. Data shown as mean ± SEM (n = 4) normalized to untreated control cells. **(p<0.01) vs BITC 30 μM (Oneway ANOVA and posthoc Dunnett T3).

We investigated whether the generation of intracellular oxidative stress is part of the mechanism by which BITC induces FOXO1 translocation and activation (Fig 4). Upon BITC treatment or BITC + insulin FOXO1 translocated to the nucleus, as previously was shown in the first translocation analysis. By contrast, when BITC was incubated in combination with N-acetyl-L-cystein (NAC) or insulin plus NAC, FOXO1 translocation into the nucleus was inhibited.

### Cell viability

**Fig 5:**
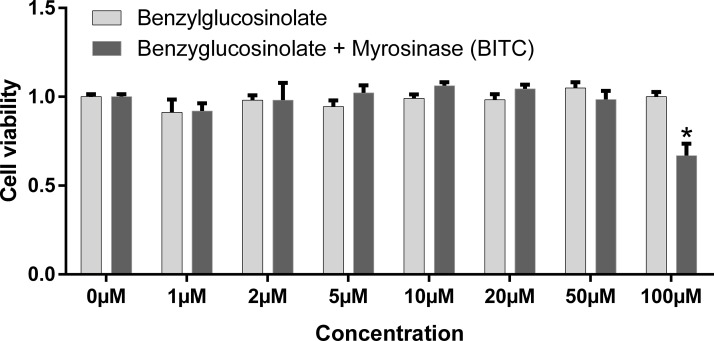
HepG2 cells survival. Following 4 h incubation at 37°C with 20 μM of PMS-electron coupling reagent (3-(4,5-dimethylthiazol-2-yl)-5-(3-carboxymethoxy phenyl)-2-(4-sulfophenyl)-2H-tetrazolium inner salt (MTS)/ phenazine methosulfate, the optical density (OD) of the MTS bioreduction product formazan was measured at 490 nm wavelength. OD mean values were normalized to mock treated cells (100% survival). Mean values + SEM (n = 3) *(p<0.05) significant differences vs control (Oneway ANOVA and posthoc Bonferroni).

The survival rates of insulin sensitive HepG2 cells expressing endogenous FOXO1 up to 50 μM BITC were not affected (Fig 5). A significant reduction of about 34% cell viability was found after 24 h for 100 μM BITC, but not for 100 μM benzyl glucosinolate. Survival of U-2 OS-FOXO1-GFP cells after the stimulation with concentrations up to 100 μM BITC was not affected, as is observed in Fig 1A–1D.

### BITC down-regulates the gene expression of G6Pase and PEPCK

**Fig 6:**
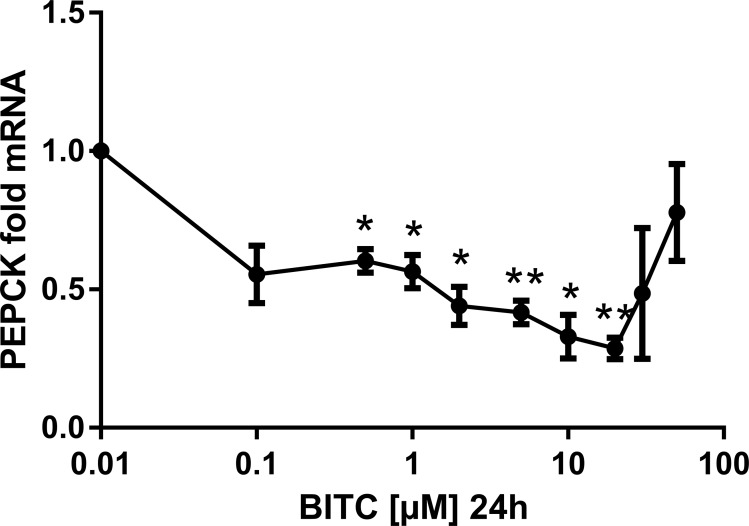
PEPCK gene expression in HepG2 cells modulated by BITC. The gene expression at selected BITC concentrations (0.1 μM to 50 μM presented in logarithmic scale) is shown for PEPCK. Fold mRNA expression was normalized to the housekeeping gene RPL32 and control (0 μM BITC), the control value is unity. One way ANOVA was used for the nonlinear regression. Data shown as mean value ± SEM of gene expression versus control *(p*<*0.05) and **(p<0.01).

**Fig 7:**
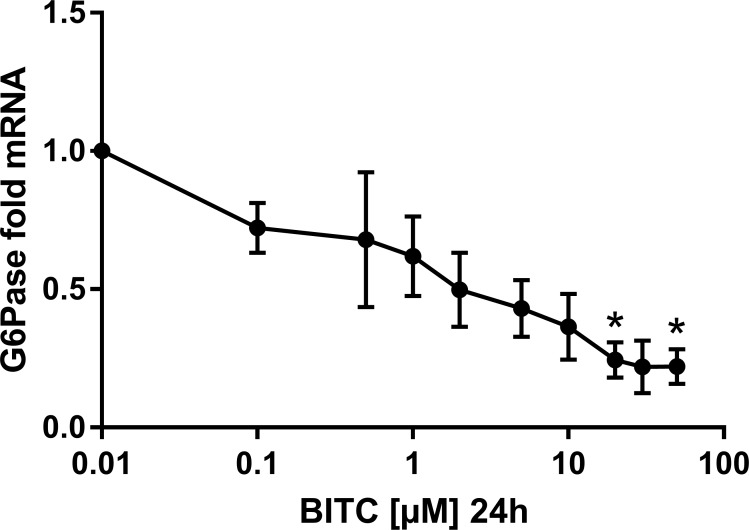
G6Pase gene expression in HepG2 cells modulated by BITC. The gene expression at selected BITC concentrations (0.1 μM to 50 μM presented in logarithmic scale) is shown for G6Pase. Fold mRNA expression was normalized to the housekeeping gene RPL32 and control (0 μM), the control value is unity. One way ANOVA was used for the nonlinear regression. Data shown as mean value + SEM of gene expression versus control *(p<0.05).

**Fig 8:**
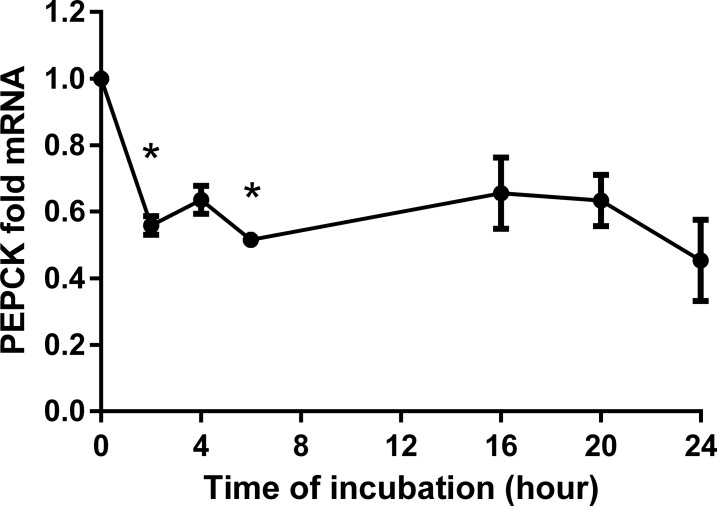
PEPCK time dependency analyses in HepG2 cells modulated by BITC. Time dependency analyses upon stimulation with BITC 30 μM is shown for PEPCK. Fold mRNA expression was normalized to the housekeeping gene RPL32 and control (0 hours), the control value is unity. One way ANOVA was used for the nonlinear regression analyses. Data shown as mean value ± SEM of gene expression versus control *(p*<*0.05).

**Fig 9:**
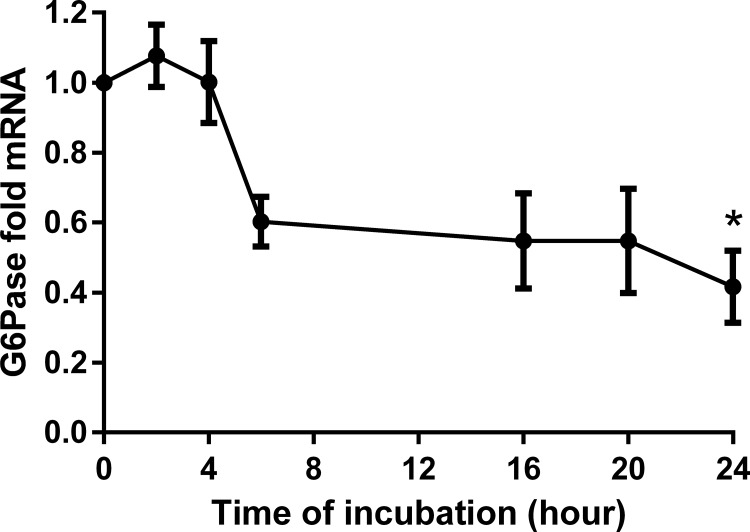
G6Pase time dependency analyses in HepG2 cells modulated by BITC. Time dependency analyses upon stimulation with BITC 30 μM is shown for G6Pase. Fold mRNA expression was normalized to the housekeeping gene RPL32 and control (0 hours), the control value is unity. One way ANOVA was used for the nonlinear regression analyses. Data shown as mean value ± SEM of gene expression versus control *(p*<*0.05).

BITC reduced markedly the gene expression of PEPCK and G6Pase in a dose dependent manner. For instance, during 24 h treatment with concentrations from 0.5 μM to 20 μM BITC, PEPCK mRNA was down-regulated significantly to 0.28-fold expression, which means 3.57-fold reduction (Fig 6). However, this effect became less pronounced and lost significance for concentrations between 30 μM and 50 μM BITC. A dose dependent reduction in the gene expression of G6Pase (significant for 20 μM and 50 μM) was achieved with 0.1 μM to 50 μM BITC (to 0.24-fold expression or 4.17-fold reduction with 20 μM (Fig 7). The effect of 30 μM BITC on the gene expression of both enzymes was continuously monitored from 2 h to 24 h (Figs 8 and 9). For PEPCK, significant differences were obtained after 2 h and 6 h (p = 0.02 and p = 0.01, respectively). For G6pase the reduction was observed from 6 h to 24 h stimulation and reached significance at 24 h (p = 0.01). The median inhibitory dose of BITC (IC50) was determined for both PEPCK (IC50 = 3.7 μM) and G6Pase (IC50 = 3.4 μM).

### BITC modulates the gene expression of antioxidant enzymes

**Fig 10:**
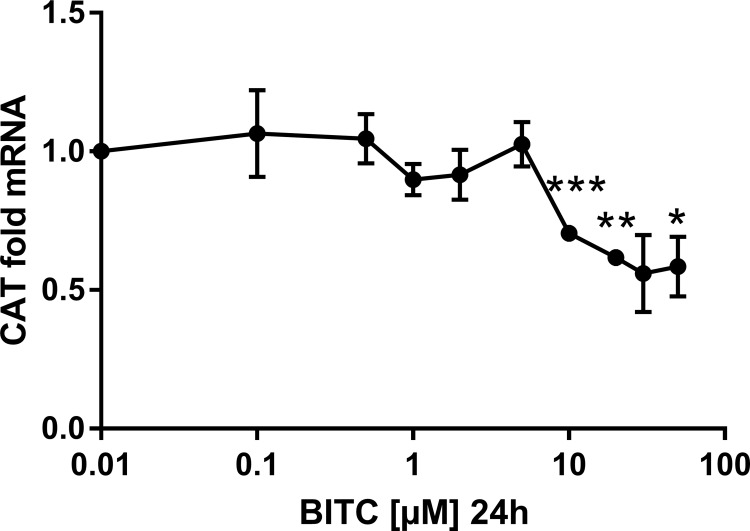
CAT gene expression in HepG2 cells modulated by BITC. Dose-dependency is shown for the antioxidant enzyme CAT. Results are presented as fold mRNA expression, normalized to the housekeeping gene RPL32 and the control. Data shown as mean value ± SEM *(p<0.05), **(p<0.01) and ***(p<0.001). (Oneway ANOVA and posthoc Bonferroni).

**Fig 11:**
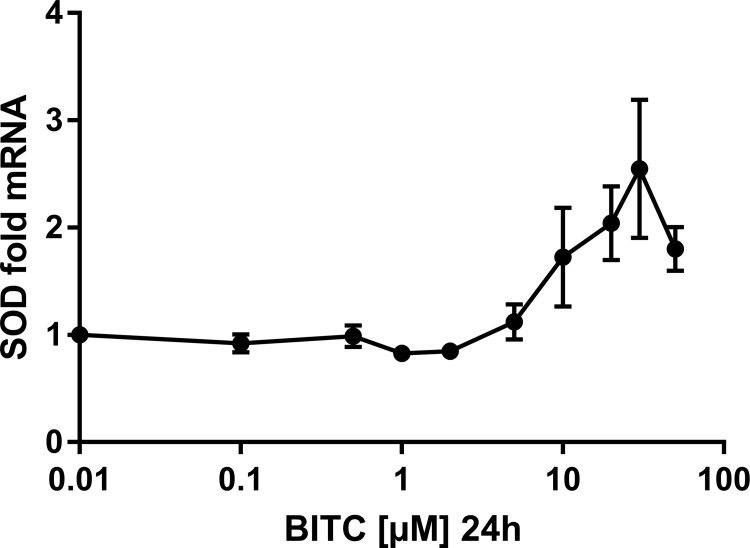
MnSOD gene expression in HepG2 cells modulated by BITC. Dose-dependency is shown for the antioxidant enzyme SOD. Results are presented as fold mRNA expression, normalized to the housekeeping gene RPL32 and the control. Data shown as mean value ± SEM *(p<0.05). (Oneway ANOVA and posthoc Bonferroni).

**Fig 12:**
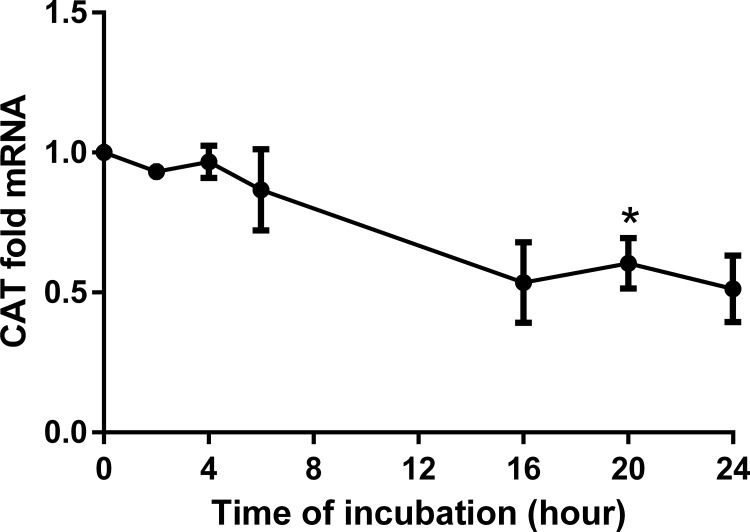
CAT time dependency analyses in HepG2 cells modulated by BITC. Time dependent analyses for CAT. Results are presented as fold mRNA expression, normalized to the housekeeping gene RPL32 and the control. Data shown as mean value ± SEM *(p<0.05). (Oneway ANOVA and posthoc Bonferroni).

**Fig 13:**
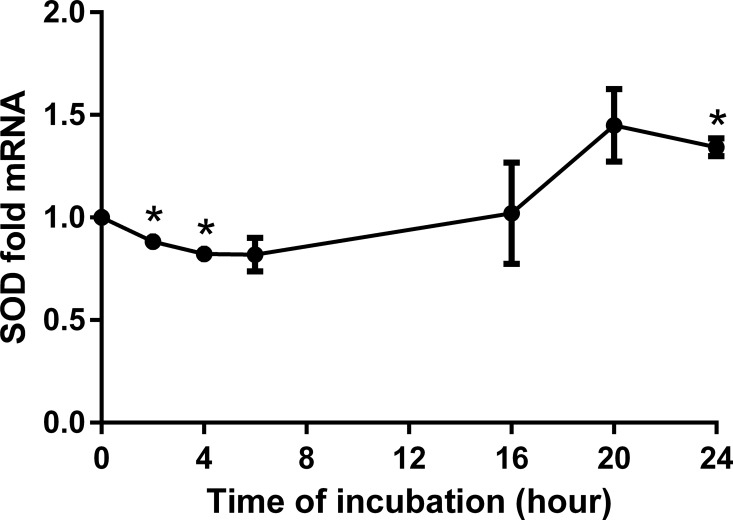
SOD time dependency analyses in HepG2 cells modulated by BITC. Time dependent analyses for SOD. Results are presented as fold mRNA expression, normalized to the housekeeping gene RPL32 and the control. Data shown as mean value ± SEM *(p<0.05). (Oneway ANOVA and posthoc Bonferroni).

BITC induced a dose-dependent down-regulation of catalase (CAT) with a significant reduction (around 50%) for concentrations higher than 10 μM (Fig 10). Conversely, manganese superoxide dismutase (MnSOD) gene expression was significantly reduced with low concentrations but increased from 10 μM BITC on, reaching a 2.5-fold maximum at 50 μM (Fig 11). More interestingly, time-dependent analyses showed that this effect just started after 16 h for both enzymes and persists up to after 24 h of incubation (Figs 12 and 13). Detoxification pathways were analyzed through gene expression analyses of nuclear factor (erythroid derived)-like2 (NRF2), Sulfiredoxin-1 (SRXN1), NAD(P)H dehydrogenase [quinone] 1 (NQO1) and Glutathione peroxidase 2 (GPX-2). HepG2 cells were treated with 30 μM BITC for 24 h and mRNA was extracted for qRT-PCR analyses. Although NRF2 is not influenced by the presence of BITC, the detoxification enzymes SRXN1 and NQO1 have shown a significant increase of 2.9- and 1.19-fold, respectively (S1 Fig).

### G6Pase and PEPCK gene expression upon FOXO1, AKT, NRF2 and SIRT1 siRNA knock-down

In order to find an explanation of the underlying mechanisms for the modulation of gene expression by BITC, the transcription factors FOXO1 and NRF2, and modulators such as AKT and SIRT1 were knocked-down and the gluconeogenic gene expression was evaluated.

**Fig 14:**
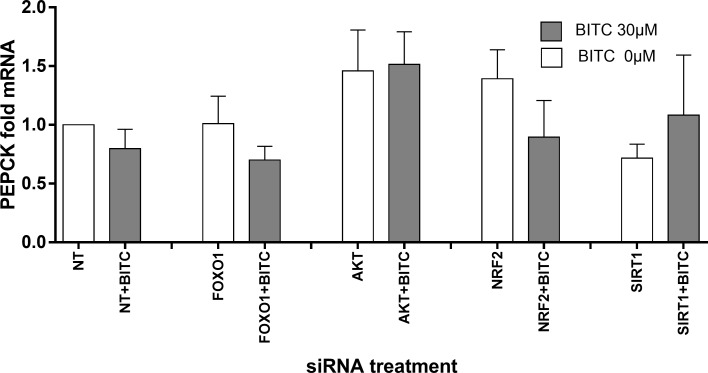
HepG2 cells siRNA knock-down analyses and PEPCK gene expression. BITC 30 μM influence after starvation on PEPCK gene expression upon FOXO1, AKT, NRF2 and SIRT knock-down conditions. (Unpaired Student’s *t* test) vs control cells treated with NT or NT+BITC, normalized with RPL32. Data shown as mean of fold mRNA + SEM (n = 4).

**Fig 15:**
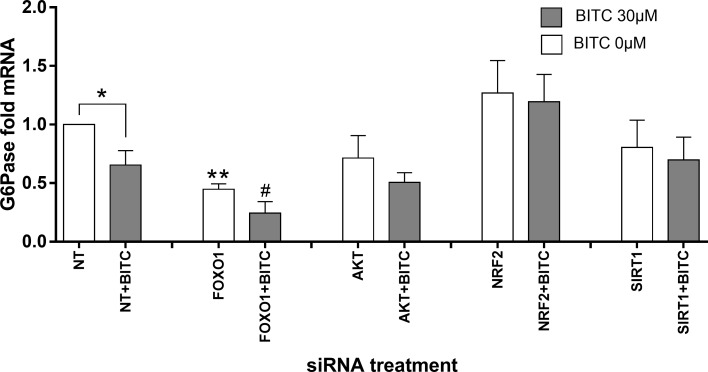
HepG2 cells siRNA knock-down analyses and G6Pase gene expression. BITC 30 μM influence after starvation on G6Pase gene expression upon FOXO1, AKT, NRF2 and SIRT knock-down conditions. *p<0.05, **p<0.01 (Unpaired Student’s t test) vs control cells treated with NT “*”or NT+BITC “#”, normalized with RPL32. Data shown as mean of fold mRNA + SEM (n = 4).

Table 2 summarizes the gene expression under FOXO1, AKT, SIRT1, NRF2 knock-down conditions. FOXO1 was efficiently knocked-down by 45% (*p* = 0.003) upon FOXO1-specific siRNA for 48 h, and its gene expression was not significantly affected by siRNA against the other factors. AKT was significantly reduced (72%, *p*<0.001) upon AKT siRNA treatment, and AKT basal gene expression was FOXO1, SIRT1 and NRF2 independent. Upon the siRNA treatment SIRT1 was successfully reduced by 43% (*p* = 0.001), and significantly increased upon FOXO1 and AKT knock-down 131% and 32%, respectively (*p* = 0.019 and *p* = 0.015, respectively). NRF2 was knocked-down by NFR2 siRNA 40% (*p* = 0.05) and not affected by FOXO1-, AKT- and SIRT1-siRNAs. None of the factors knock-down had significant effects on PEPCK gene expression (Fig 14), although under AKT knock-down a non-significant increase of PEPCK mRNA was observed indicating a higher expression rate under reduced AKT leading to lower phosphorylating capacity for inactivation of FOXO1. G6Pase mRNA was reduced by 55% upon FOXO1 knock-down (p = 0.007, Fig 15), which confirms the role of FOXO1 in regulating G6Pase gene expression. Upon BITC treatment for 24 h, G6Pase mRNA was significantly down-regulated 35% in control cells (p<0.05) and 62% upon FOXO1 knock-down (p<0.05) compared to cells without siRNA treatment and stimulated with BITC. In the last case the significance reached by BITC compared to the unstimulated cells was reduced to less than half of the expression rate achieved under FOXO1 knock-down. An increasing trend in PEPCK and G6Pase basal gene expression upon NRF2 knock-down was observed, and after BITC stimuli the down-regulation of G6Pase mRNA was abolished compared to cells without siRNA treatment and stimulated with BITC.

**Table 2:** Gene expression changes (%) after FOXO1, AKT, SIRT1 and NRF2 knock-down.

si RNAs Treatment	FOXO1 mRNA Levels	AKT mRNA Levels	SIRT1 mRNALevels	NRF2 mRNALevels
Control (NT)	100	100	100	100
FOXO1	**-45,30	+27,33	*+131,81	+155,80
AKT	-4,72	***-71,90	*+32,58	+67,45
SIRT1	+33,19	+27,12	***-42,71	+96,21
NRF2	+37,45	+25,58	+64,24	*-39,77

48 h after transfection of HepG2 cells, including a starvation period of 16 h in EMEM without FBS, RNA was extracted, reverse transcribed and qRT-PCR performed with primers given in Table 1. Quantified relative amplification vs housekeeping gene RPL32 expression was normalized to the rate in HepG2 transfected with non-target NT siRNA (100% rate of expression). Mean mRNA levels (n≥ 4) are shown as percentage of change of basal expression regarding to the transfection controls. The knock-down efficiencies for each factor are shown in grey. The statistical significance level was set at p<0.05 (Unpaired Student’s *t* test). *p<0.05, **p<0.01 and ***p<0.001 vs control cells transfected with Non Target siRNA (NT) normalized with RPL32 and control.

### Effect of BITC on phosphorylation and protein expression

#### i) AKT- and FOXO1 phosphorylation

**Fig 16:**
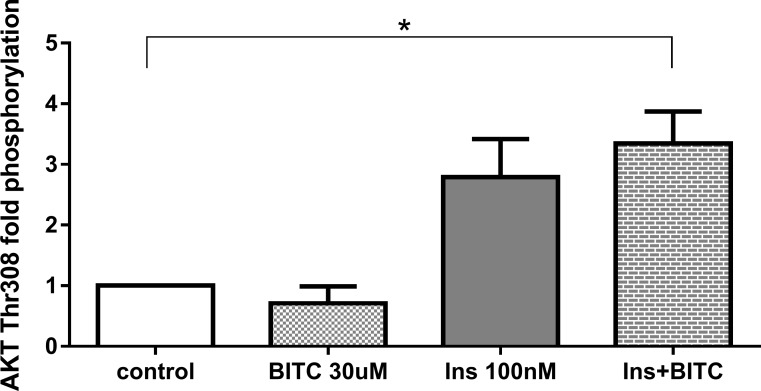
AKT (Thr308) phosphorylation upon BITC and insulin treatment. HepG2 cells were incubated with BITC 30 μM, insulin 100 nM and insulin + BITC for 30 min. PathScan Signaling Array Kit was used for the detection of the phosphorylated AKT (Thr308). Data shown as mean of fold phosphorylation normalized to untreated control + SEM (n = 3) *p<0.05(Unpaired Student’s t test).

**Fig 17:**
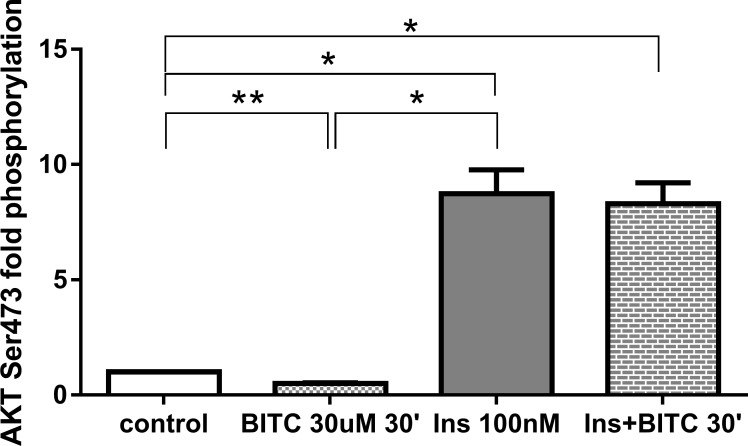
AKT (Ser473) phosphorylation upon BITC and insulin treatment. HepG2 cells were incubated with BITC 30 μM, insulin 100 nM and insulin + BITC for 30 min. PathScan Signaling Array Kit was used for the detection of the phosphorylated AKT (Ser473). Data shown as mean of fold phosphorylation normalized to untreated control + SEM (n = 3) *p<0.05, **p<0.01 (Unpaired Student’s t test).

**Fig 18:**
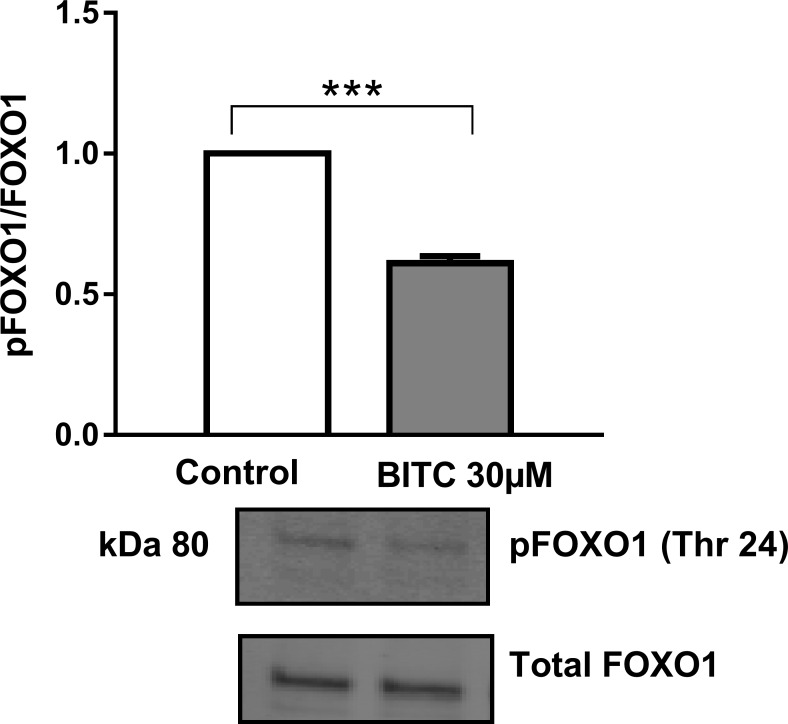
FOXO1 phosphorylation upon BITC treatment. HepG2 cells were incubated with 30 μM BITC for 24 h and cell lysates were prepared and subjected to Western blot analysis with antibodies for pFOXO1 Thr24 (Phospho FOXO1). Total FOXO1 was used for normalization. Data shown as mean + SEM (n = 3) ***p<0.001 (Unpaired Student’s t test).

Path scan analyses were performed to elucidate the mechanisms, as post-translational modifications, by which BITC modulates FOXO1 translocation and activation. As expected, incubation with insulin 100 nM increased AKT phosphorylation, which was no reversed by BITC for Ser473 and for Thr308 (Fig 16) when BITC was incubated after insulin stimuli. Treatment of HepG2 cells with 30 μM BITC for 30 min induced a significant reduction in the phosphorylation of AKT at Ser473 (Fig 17). Western blot analyses evaluating FOXO1 phosphorylation following a pre-incubation with 30 μM BITC for 24 h showed a significant reduction in pFOXO1 as compared to untreated cells (Fig 18).

#### ii) MAPKs protein phosphorylation

**Fig 19:**
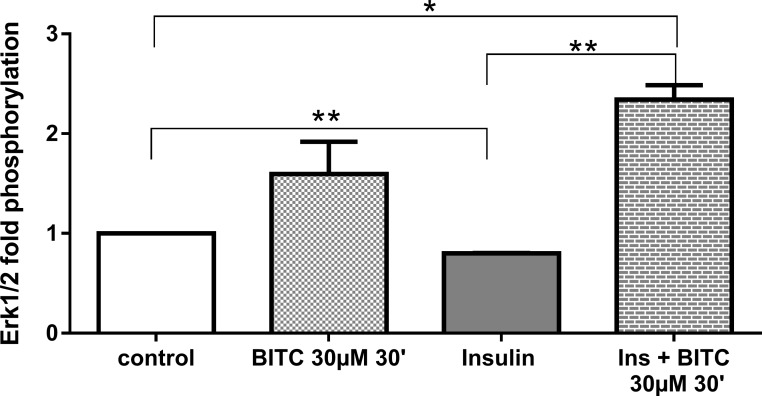
ERK phosphorylation upon BITC and insulin treatment. HepG2 cells were incubated with BITC 30 μM, insulin 100 nM and insulin + BITC for 30 min. PathScan Signaling Array Kit was used for the detection of the phosphorylated molecule ERK1/2(Thr202/Tyr204). Data shown as + SEM (n = 3) * p<0.05, **p<0.01 (Unpaired Student’s *t* test).

**Fig 20:**
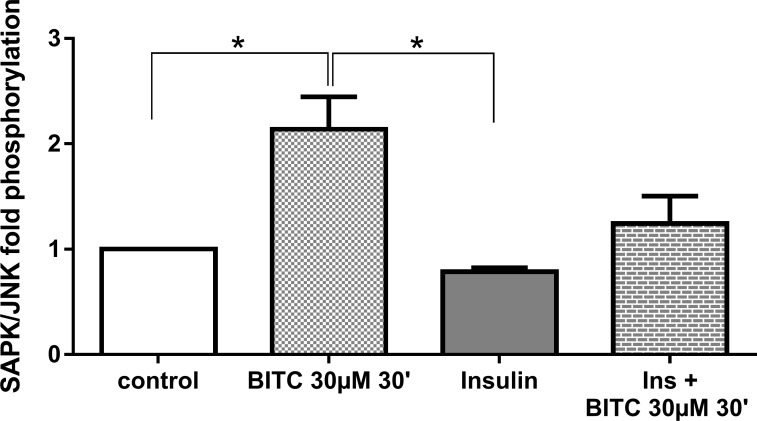
JNK phosphorylation upon BITC and insulin treatment. HepG2 cells were incubated with BITC 30 μM, insulin 100 nM and insulin + BITC for 30 min. PathScan Signaling Array Kit was used for the detection of the phosphorylated molecule SAPK/JNK (Thr183/Tyr185). Data shown as + SEM (n = 3) * p<0.05 (Unpaired Student’s *t* test).

Although not significant, BITC increased ERK 1/2 phosphorylation 1.5 fold (Fig 19), as compared with control cells. BITC also induced by >2 fold the phosphorylation when cells where stimulated in combination with insulin (p = 0.02). JNK phosphorylation was significant increased 2.13 fold after the stimulation with BITC (Fig 20). BITC reversed the inhibitory effect of insulin on JNK phosphorylation when the cells where treated with insulin and BITC together, in comparison with cells treated only with insulin.

#### iii) Gluconeogenic proteins

**Fig 21:**
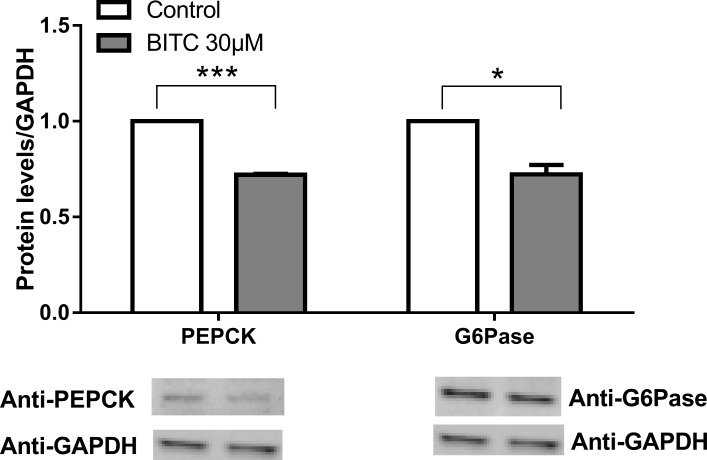
Gluconeogenic enzymes protein expression. HepG2 cells were incubated with 30 μM BITC for 24 h and cell lysates were prepared and subjected to Western immuno-blot analysis with primary antibodies for PEPCK and G6Pase. Results are normalized with GAPDH. Data shown as + SEM (n = 3) **p<0.01, ***p<0.001 (Unpaired Student’s *t* test).

A significant reduction in PEPCK and G6Pase protein expression occurred after treatment with BITC p<0.001 and p<0.01 respectively (Fig 21). The results are in agreement to those observed in the gene expression analyses (Figs 6 and 7).

## Discussion

This work has shown that in U-2 OS cells stably expressing FOXO1-GFP BITC is able to promote FOXO1 nuclear accumulation in a dose dependent manner preventing FOXO1 degradation in the cytoplasm. These results are supported by previous studies from Boreddy *et al*. who showed an increased FOXO1 nuclear accumulation and FOXO1 dependent transcription upon BITC stimulation by immunofluorescence analysis in pancreatic cells [32]. In contrast, in our study FOXO1 translocation was completely inhibited in U-2 OS cells pretreated with the antioxidant NAC, suggesting that induction of oxidative stress by BITCs is required for the FOXO1 translocation, as also shown for lung cancer cells [33]. However, Zhang indicates that NAC stimulation might block the uptake of BITC in murine hepatoma cells [34]. It is possible that in our work the observed NAC effect of cytoplasmic retention of FOXO1 may, in part, be due to an inhibition of BITC uptake.

The oxidative stress induction and its effect on FOXO1 nuclear import and accumulation have been demonstrated by other phytochemicals. Recent studies show that resveratrol behave as a pro-oxidizing agent [35] exerting its favourable biological effects through the induction of intracellular reactive oxygen species, promoting FOXO1 nuclear import and activation, which induce the transcription of factors involved in antioxidant pathways [11]. Thus the activation of FOXO1 induces cellular protective pathways such as oxidative stress resistance, apoptosis and autophagy [36, 37]. Although, in T2D oxidative stress is currently considered to play a role in diabetic micro- and macrovascular complications, the induction of antioxidative mechanisms might improve the cellular defense, depending on the pro- and antioxidative redox balance. It is important to highlight that although the FOXO1 nuclear import, FOXO1 accumulation and SIRT1 gene expression induction observed in the present study are critical steps in FOXO1 transcription induction, it was not evaluated in the current study.

FOXO1 nuclear accumulation could additionally be the result of a reduction of its phosphorylation by AKT (the main pathway of FOXO nuclear exclusion and degradation following insulin or IGF signaling) or by ERK [38]; dephosphorylation by protein phosphatases [39]; or additional post-translational modifications such as phosphorylation by JNK [40], p38 [41] or cyclin dependent kinase 1 (CDK1) [42], which have been described as inducers of FOXO1 nuclear accumulation in response to oxidative stress and other stimuli [10, 43].

Insulin was able to antagonize the effect of BITC, since incubation with BITC in combination with insulin induced FOXO1 accumulation. Since FOXO1 protein was located in the cytoplasm and migrated into the nucleus upon BITC stimulation, a reduced level of pFOXO1 was expected after BITC stimulus. The effect of 30 μM BITC on pFOXO1 levels was evaluated by stimulating the cells for 24 h similar to the procedure used for the gene expression analyses, and a significant reduction of pFOXO1 was observed. The reduction in FOXO phosphorylation upon isothiocyanates stimulation has been shown previously by Boreddy *et al*. in mice pancreatic cancer cells [32] and by Davis *et al*. in human umbilical vein endothelial cells [44].

The reduction in pAKT (Ser473) by BITC (Fig 17) confirms the role of the AKT pathway in the regulation of FOXO1 translocation upon the BITC stimulus shown by others previously [32]. The MAPK, ERK, and JNK proteins activated by stress stimuli [45, 46], were evaluated to address additional mechanisms involved in the FOXO1 activation. JNK, which has up-regulated significantly in the present study, is responsible for FOXO1 modulation [41, 47]. JNK induces FOXO1 nuclear import enhancing the glucose production in hepatitis C virus-infected cells [40] or enhancing angiogenesis in fibroblasts [41]. ERK in contrast promotes FOXO1 nuclear export [41], by phosphorylation in nine serine residues, different from those three residues that can be phosphorylated by AKT which were not evaluated in this study [13]. The FOXO1 translocation and nuclear accumulation observed in the current study suggests a probable inducing effect by JNK. We could assume that the non-significant increase in ERK phosphorylation could be linked to the inhibition in gluconeogenesis found in this work. Previous studies have reported that activation of the MAPK/ERK pathway can also inhibit the expression of PEPCK and G6Pase genes, most likely through a mechanism independent from insulin [13]. The inducer effect of ITCs on MAPKs has also been demonstrated previously in the prevention of cancer in other studies [46].

FOXO transcription factors regulate gene expression positively and negatively [12]. Previous studies provide evidence that in the absence of insulin stimulation or in the fasting state, FOXO1 induces glucose production through the induction of G6ase and PEPCK gene expression [48]. In this study BITC induces a dose dependent down-regulation of gluconeogenesis, as confirmed by the reduction in PEPCK and G6Pase gene and protein expression. BITC reduced PEPCK mRNA within the first 6 h of stimulation (early phase of gluconeogenesis), while G6Pase was down-regulated from 6 h to 24 h (late phase of gluconeogenesis), revealing BITC to act as an inhibitor of gluconeogenesis. This could be considered as a positive factor in T2D prevention or treatment, since increased hepatic glucose production is a major feature of the disease [49] and an inhibition of gluconeogenesis could improve directly or indirectly insulin signaling and sensitivity. Our suggestion is supported by Waterman C *et al*. who found a reduction in gluconeogenic enzymes gene expression in liver of mice fed with very high fat diet supplemented with moringa benzyl isothiocyanate [25]. *In vivo* studies in humans support also our results, Bahadoran *et al*. showed a significant reduction in fasting glucose and insulin resistance after a 4-week-treatment of type 2 diabetic patients with 5 and 10 g/d broccoli sprouts powder containing mainly 4-methylsulfinylbutyl glucosinolate [50].

In agreement with the siRNA analyses, the BITC effect on gluconeogenic genes is independent from all the factors knocked-down. Despite not significant higher expression of PEPCK was observed under reduced AKT. Less AKT can be responsible for reduced FOXO1-phosphorylation under AKT siRNA conditions, which could further induce PEPCK gene expression; however, this effect was not disturbed by BITC in the present study. Compared to NT-siRNA treatment, FOXO1 modulation of G6Pase expression is demonstrated by the reduction of G6Pase after FOXO1-siRNA. The significant down-regulation by BITC could be considered FOXO1-independent, as the significance in FOXO1-siRNA treated cells is not lost. Only NRF2 seems to be necessary for the effect of BITC-induced G6Pase-reduction, as the significant inhibitory effect of BITC on G6Pase was abolished after NRF2-siRNA knockdown at a higher level of G6Pase expression compared to NT-siRNA treatment. Recent promoter analysis of human PEPCK performed by our group showed 10 binding motifs for NRF2, one of them near to the transcription start site close to the FOXO1-binding site IRE2 [15]. Aleksunes *et al*. showed an increase in G6Pase gene expression in Nrf2-null mice, indicating that Nrf2 influences the transcriptional regulation of gluconeogenic genes [51]. The potential NRF2 effect on gluconeogenesis regulation is supported by Slocum *et al*., which recently showed that increased Keap1/Nrf2 signaling in the liver of hypomorphic Keap1 allele mice is accompanied by repressed gluconeogenesis, measured by a reduction in PEPCK and G6Pase gene expression [52].

It is noteworthy that PEPCK and G6Pase are regulated both directly and indirectly via transcriptional and posttranscriptional mechanisms in a network type manner [53], which can mutually compensate their functions in the absence or reduction of each single factor, making it difficult to determine the relative contribution of individual transcription factors to these processes [54]. These results suggested that the AKT-FOXO1 cascade might not be the only driver of the transition from fasting to feeding metabolic states [55]. FOXO1 transcriptional activity has been related to the late phase of gluconeogenesis [56], were it interacts with other transcription factors such as PGC-1α, ERRγ and HNF4 inducing G6Pase and PEPCK gene expression [48]. In the current study the BITC dependent down-regulation in PEPCK and G6Pase was observed within the first 6 h and up to 24 h, respectively, which suggests that additional factors different from FOXO1 could be involved in the gluconeogenic response to BITC. Recently, FOXO6 was also found to be involved in gluconeogenesis [57], suggesting that in the control of hepatic gluconeogenesis *in vivo* some redundancy might be present among FOXO transcription factors [48]. The progression and complications of diabetes are linked to oxidative stress [5] through the glucose auto-oxidation, overproduction of ROS by mitochondria, non-enzymatic glycation, and the polyol pathway [3]. FOXO transcription factors are critical regulators of cell fate and play a major role in the oxidative stress responses modulating antioxidant and detoxification enzymes in order to protect the cells from ROS [7, 58, 59]. Owing to antioxidant enzymes CAT and SOD and detoxification proteins NQO1, SFRXN and GPX-2 have an established role in the oxidative stress resistance and have been linked with FOXO1 activity as well [60], therefore, the potential modulatory effect of BITC on their gene expression was evaluated in the frame of this work.

CAT has been described as a FOXO1 target gene, which is induced after FOXO1 activation [60, 61]. In this study BITC reduced significantly CAT gene expression in a dose dependent manner after 16h incubation, suggesting that another unknown pathway affected by BITC is eventually regulating CAT gene expression. Similar results have been shown by others in liver cells after the stimulation with resveratrol [35].

According to the analyses done in the present study, BITC had a two-phase effect in SOD gene expression. It starts with a significant reduction (*p =* 0.01) at low doses of BITC (1 μM and 5 μM), followed by a dose dependent up-regulation at higher concentrations (10–50 μM). The down-regulation in SOD could be comparable to that reported by Zhu Y. *et al*. who found a reduction of SOD activity upon the stimulation with BITC (2 μM and 5 μM) after 24h [62]. Up to the current date there are no reports evaluating the gene expression of SOD upon high concentrations of BITC. The results suggest that SOD might be up regulated as a compensatory response to the oxidative stress conditions produced by BITC, as observed in preliminary studies [62, 63].

The detoxification enzymes NQO1 and SRXN1 were up-regulated upon BITC stimulation. Similar results have been reported for the isothiocyanate sulforaphane, known for its NRF2 dependency in the induction of detoxification enzymes [64]. Sulforaphane promotes NRF2 nuclear import by the interaction with its cytoplasmic repressor kelch-like ECH-associated protein 1 (KEAP1). In the nucleus NRF2 heterodimerizes with small Maf (V-maf musculoaponeurotic fibrosarcoma oncogene homolog) proteins and binds to antioxidant response elements (AREs), inducing the expression of several cytoprotective genes [64]. These facts suggest that the effect of isothiocyanates including BITC on NRF2 modulation are regulated at post-translational levels, which could explain the lack of effects on NRF2 gene expression upon BITC stimulus.

It can be concluded from the previous results that BITC might act in the cell as a pro-oxidant agent, triggering an antioxidant effect only at high concentrations, as can be deduced by the induction in SOD, SFRXN and NQO1 gene expression after the stimulation with 30μM BITC. As shown for other isothiocyanates [65], the pro-oxidant effect of BITC could induce some gene and protein expression alterations through the activation of antioxidant defense systems, which could result in cell survival and adaptation [35]. However, it is clear that each antioxidant is in fact a redox agent and thus might become pro-oxidant under special conditions; this pro-oxidant action might not always be adverse for humans [35]. It has been proposed that the pro-oxidant action of antioxidant vitamins and several classes of plant-derived polyphenols rather than their antioxidant activity, might play a crucial role in their anticancer and apoptosis inducing properties [32, 66, 67]. During normal diet consumption, most of plant-derived bioactive compounds would not reach toxic concentrations. Nevertheless, the concentrations apparently reached *in vivo* are sufficient to induce the activation of intracellular protective signaling pathways that have evolved to recognize potential threats by sub-toxic concentrations of pro-oxidant bioactive compounds or electrophiles [67].

Altogether, the present study provides comprehensive insights into the effect of BITC on the PI3K/AKT/FOXO1 pathway. BITC induces FOXO1 nuclear import and accumulation, probably through the inhibition of AKT and FOXO1 phosphorylation. The FOXO1 nuclear accumulation allows FOXO1 to induce oxidative stress resistance and detoxification. It could be concluded that the gene expression of factors described as FOXO1 target genes is modulated by additional factors in absence of FOXO1. BITC can modulate the gene expression of FOXO1 target genes by targeting, probably, other FOXO proteins, which can compensate their function under knock-down conditions.

These results show that BITC mimics the fasting state, in which the insulin stimulus is absent and FOXO proteins remain in the nucleus modulating gene expression of their target genes. Although in the present study the gene expression of gluconeogenesis, antioxidant and detoxification proteins was FOXO1 independent, one could assume that BITC targets additional transcription factors responsible for gluconeogenesis inhibition and detoxification. Since in insulin resistant conditions insulin is not able to suppress gluconeogenesis, the inhibition of G6pase and PEPCK by BITC might be considered positive in type 2 diabetes prevention and treatment. Therefore, the factors behind its modulatory effects merit further investigation. Since the final purpose of the search of bioactive compounds from plants for chronic disease prevention is their consumption by humans, the effects of BITC on T2D prevention should be considered in *in vivo* studies.

## Supporting Information

S1 Fig Detoxification enzymes gene expression in HepG2 cells modulated by BITC. Results are presented as fold mRNA expression, normalized to the housekeeping gene RPL32 and the control. Data shown as + SEM (n = 3) *p<0.05 (Unpaired Student’s t test).
(TIF)

S1 Video Live cell imaging of FOXO1-GFP translocation induced by BITC in U-2 OS. U-2 OS FOXO1-GFP cells were incubated with 50 μM BITC for 1 h, the time-dependence FOXO1 translocation was evaluated by Live cell Imaging.
(WMV)
