# Supplementary figures and images for: Benzylglucosinolate Derived Isothiocyanate from Tropaeolum majus Reduces Gluconeogenic Gene and Protein Expression in Human Cells
Source: PLoS One. 2016 Sep 13;11(9):e0162397. doi: 10.1371/journal.pone.0162397 (PMC5021297; doi:10.1371/journal.pone.0162397)

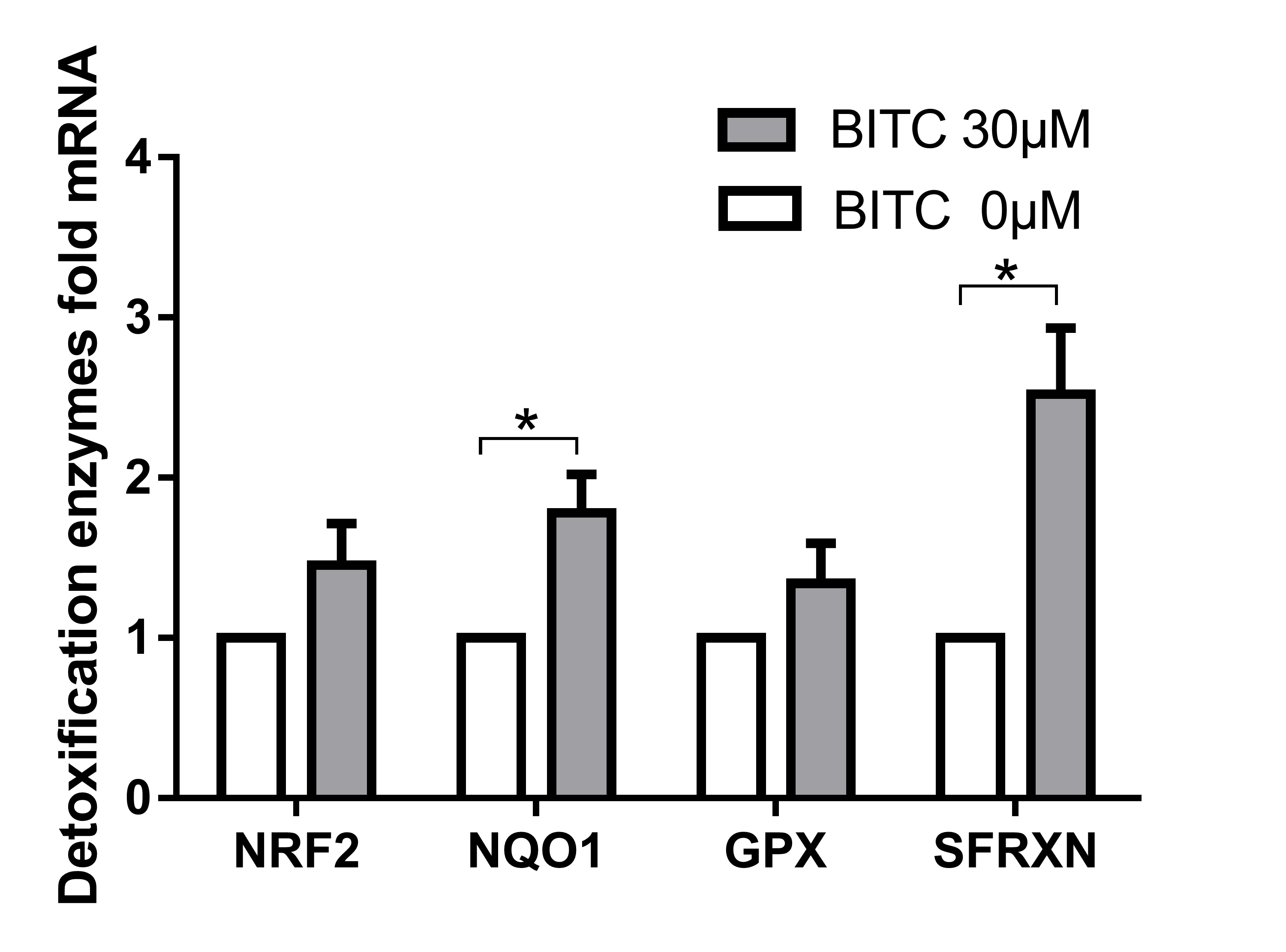

Supplement: S1 Fig — Results are presented as fold mRNA expression, normalized to the housekeeping gene RPL32 and the control. Data shown as + SEM (n = 3) *p<0.05 (Unpaired Student’s t test). (TIF) [file pone.0162397.s001.tif]
